# Assessing the impact of including sterile 2% chlorhexidine gluconate with 70% isopropyl alcohol applicator in a preoperative skin preparation bundle on surgical siteinfection rate

**DOI:** 10.1017/ash.2025.91

**Published:** 2025-09-03

**Authors:** Kristin Hui Xian Tan, Ning-Ling Huang, Yan Ma

**Affiliations:** 1 Becton Dickinson Holdings Pte Ltd, Singapore Abstract topic: Healthcare Associated Infection (HAI), Singapore

## Abstract

**Objectives:** 2% chlorhexidine gluconate (CHG) with 70% isopropyl alcohol (IPA) has been recommended over povidone-iodine (PVI) for skin preparation. BD ChloraPrep™ is a ready-to-use applicator pre-filled with sterile 2% CHG and 70% IPA solution, which healthcare professionals have reported higher preference over PVI in an applicator or a bulk bottle. This study aims to evaluate the impact of including 2% CHG with 70% IPA applicator in the care bundle for preoperative skin preparation on surgical site infection (SSI) rate. **Methods:** A systematic literature review was conducted on PubMed in July 2022. Study inclusion criteria were (1) English publications, (2) 2% CHG with 70% IPA applicator included in a preoperative skin preparation bundle and (3) SSI rate reporting. A weighted average of SSI rate change was calculated, using the study sample size as weights. **Results:** A total of 116 studies were identified and 51 of them were found relevant for further review. Of them, 18 studies met the study inclusion criteria and 13 of these publications (72%) studied BD ChloraPrep™ in their bundle of care. 92% (12/13) of these studies demonstrated a statistically significant reduction in SSIs. 10 studies reported statistically significant SSI reduction rates, one study reported full compliance with the care bundle was associated with lower risk of SSI and one study reported four-fold higher likelihood of achieving zero SSI. Based on the 10 studies which reported statistically significant SSI reduction rates, a sample-size-weighted average of 71% reduction in SSI was observed when BD ChloraPrep™ was included in the bundle for preoperative skin preparation. **Conclusions:** There remains considerable low compliance and major variation in standardised skin preparation practices among hospitals. Using BD ChloraPrep™ may encourage a standardised and thorough approach in skin preparation. With the implementation of a bundle, together with appropriate training, compliance with skin preparation may be improved, which can potentially reduce SSIs.

